# Differential activity of 16K rat prolactin in different organic systems

**DOI:** 10.1080/19768354.2018.1554543

**Published:** 2019-02-20

**Authors:** Bo-Young Yun, Chunghee Cho, Byung-Nam Cho

**Affiliations:** a Department of Life Science, The Catholic University of Korea, Bucheon, Korea; b Department of Life Science Gwangju Institute of Science and Technology, Gwangju, Korea

**Keywords:** 16K prolactin, anti-angiogenesis, WBC proliferation, reproduction

## Abstract

The 16K isoform of rat prolactin (16K rPRL) performs multiple functions in various systems including angiogenesis, tumorigenesis, and reproduction. Recently, 16K rPRL has attained prominence as a possible therapeutic target in pathophysiological conditions. However, the integral function and mechanism of 16K rPRL in various systems has not been elucidated. To this end, a transient gain-of-function animal model was adopted. An expression DNA plasmid containing 16K rPRL or rPRL gene was introduced into the muscle of adult mice by direct injection. The mRNA and protein expression levels of 16K rPRL were detected by initial RT–PCR and subsequent Southern blot and western blot, respectively. When the expression vector was introduced, the results were as follows: First, 16K rPRL combined with rPRL reduced angiogenesis in the testis whereas rPRL alone induced angiogenesis. Second, 16K rPRL combined with rPRL reduced WBC proliferation, whereas rPRL alone increased WBC proliferation. Third, 16K rPRL combined with rPRL reduced diestrus, whereas rPRL alone extended diestrus. Fourth, 16K rPRL combined with rPRL unexpectedly increased testosterone (T) levels, whereas rPRL alone did not increase T levels. Taken together, our data suggest that the 16K rPRL isoform performs integral functions in angiogenesis in the testis, WBC proliferation, and reproduction, although the action of 16K rPRL is not always antagonistic.

## Introduction

The 16K PRL is a 16-KDa N-terminal fragment of 23K prolactin (PRL). PRL is a peptide hormone synthesized and secreted by the lactotroph cells of the anterior pituitary (Maurer 1980). Synthesis of PRL is not, however, limited to the pituitary, implying other roles for PRL, as numerous extra-pituitary sites of PRL expression have been identified, including the placenta (Lee and Markoff 1986), lymphocytes (Pellegrini et al. 1992), and breast cancer cells of epithelial origin (Ginsberg and Vonderhaar 1995). The 16K PRL isoform is produced as a result of proteolytic cleavage of PRL by cathepsin D, matrix metalloproteinases, or bone morphogenic protein (Clapp et al. 2006). The 16K PRL isoform has inhibitory effects on angiogenesis and tumorigenesis, whereas the major PRL isoform is involved in lactation and reproduction (Bernard et al. 2015). A more precise understanding of the function and mechanism of 16K PRL in physiological and pathological conditions is required.

The 16K PRL isoform was initially known to have an anti-angiogenic role. In fact, 16K PRL and related PRL family proteins including human GH, GH variants, and placental lactogen, have been found to be anti-angiogenic (Clapp et al. 1993; Struman et al. 1999). It is known that 16K PRL levels are increased in retinopathy (Garcia et al. 2008) and postpartum cardiomyopathy (Hilfiker-Kleiner et al. 2007), diseases that are accompanied by angiogenesis or anti-angiogenesis. As to PRL, PRL and PRL-related proteins have been found to play a role in angiogenesis (Clapp et al. 1993; Jackson et al. 1994), a vital aspect of many physiological processes including wound healing and organ regeneration (Hanahan and Folkman 1996) as well as of pathological conditions such as tumor growth and metastasis (Folkman 1995). In an ectopic PRL expression model, introduced PRL induced angiogenesis in the testis (Ko et al. 2003; Lee et al. 2006). One controversial result reported in the literature is that intact rPRL had no effect on angiogenesis (Ferara et al. 1991). In addition, proliferin, a PRL-related protein, stimulated angiogenesis, whereas proliferin-related protein inhibited it (Jackson et al. 1994).

The role of 16K PRL in relation to WBC proliferation is not well known. It has been reported only that PRL is an *in vitro* co-mitogen for T and B cells of human or murine origin (Russell et al. 1984; Bernton et al. 1988; Clevenger et al. 1990; Ko et al. 2003). Controversial results that contradict these findings have also been reported (Gala and Shevach 1997). PRL regulates lymphocyte proliferation by modulating the expression of gene products necessary for cell cycle regulation (Clevenger et al. 1992) via the T and B lymphocyte PRL receptor (Pellegrini et al. 1992). Recently, transgenic mice have been generated that overexpress PRL (Wennbo et al. 1997), as well as others with targeted disruptions of PRL (Horseman et al. 1997) or the PRL receptor (Ormandy et al. 1997; Bouchard et al. 1999). However, relatively little information is presently available about these mice. Consequently, many long-standing controversies regarding the role of PRL in hematopoietic processes remain unclear. Moreover, the role of 16K PRL in hematopoietic processes, including WBC proliferation, remains unknown.

With respect to reproduction, PRL is well known to participate in regulation of reproduction (Leong et al. 1983), osmoregulation (Neill 1988), and immununomodulation (Bole-Feysot et al. 1998); however, our knowledge of the role of 16K PRL in postpartum cardiomyopathy (Hilfiker-Kleiner et al. 2007) and the onset of preeclampsia (Gonzalez et al. 2007) is limited. In females, PRL is known for its action on ovarian function. The luteotropic and luteolytic actions of PRL have been recognized for a number of years in rodents. In general, the luteotropic action of PRL involves stimulation of progesterone production by luteal cells (Matsuyama et al. 1990). In mammals, depending on the stage of the cycle, the luteolytic effects of PRL have also been reported (Loudon et al. 1990). Our previous report revealed that ectopic PRL expression extended the diestrus stage, resulting in extension of the estrous cycle, an important phenomenon in reproduction (Ko et al. 2003; Lee et al. 2006). Our knowledge of the physiological role of PRL in males is limitedl. The absence of PRL signaling in PRL-receptor deficient mice is not detrimental to male testicular function and to fertility (Binart et al. 2003) although PRL increases LH receptor numbers (Dombrowicz et al. 1992), steroidogenesis (Gunasekar et al. 1988) in Leydig cells, and angiogenesis in the testis (Ko et al. 2003; Lee et al. 2006).

## Materials and methods

### Animals and experimental design

**Figure 1.**
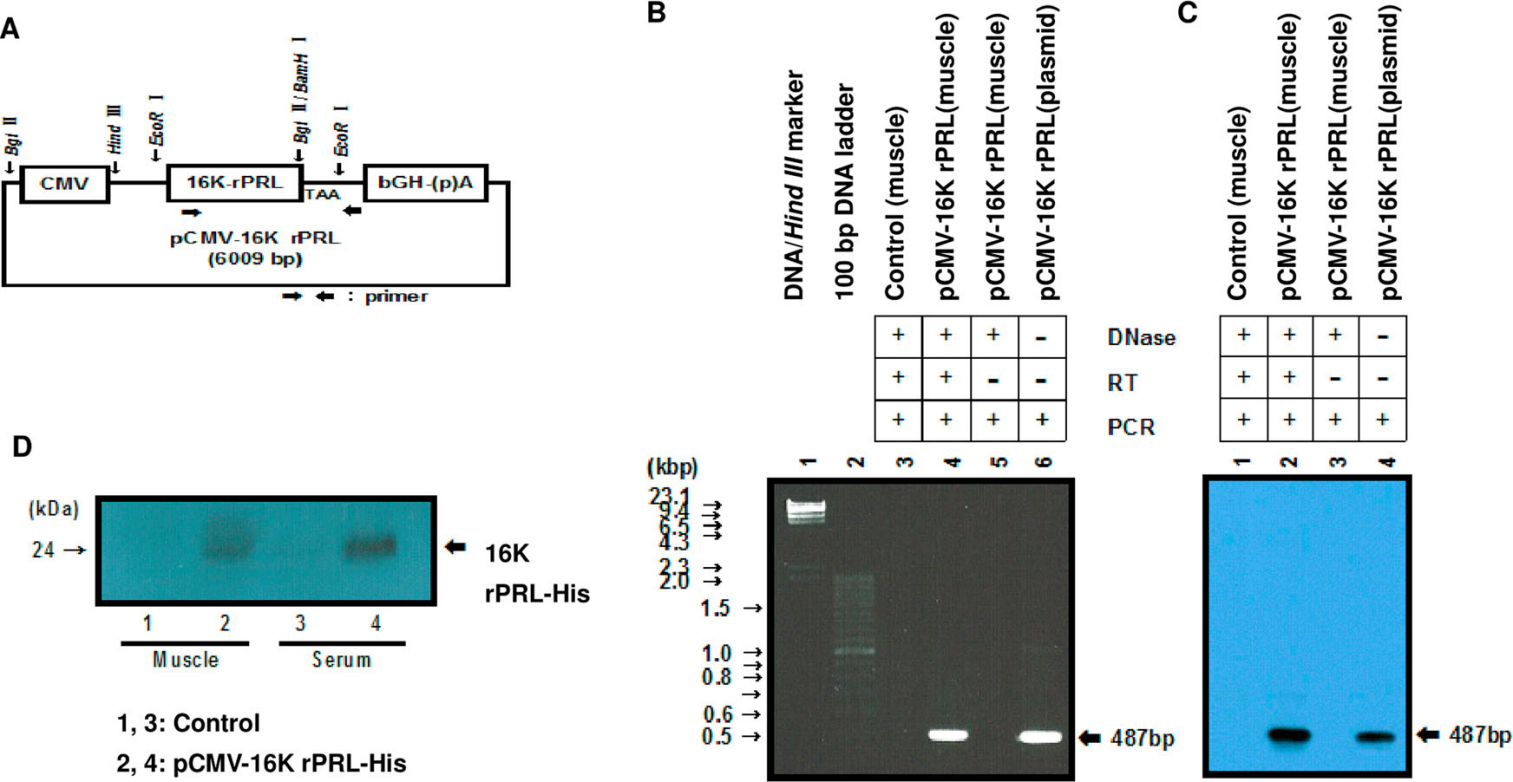
pCMV-16K rPRL structure and 16K rPRL expression. A. Diagram of the pCMV-16K rPRL construct. Functional elements include the cytomegalovirus (CMV) promoter, rat PRL cDNA, and human growth hormone (hGH) poly (A). B-C. RT-PCR (B) and Southern blot analysis (C) were performed as described in Materials and Methods. RNAs from control and pCMV-16K rPRL-injected mice without reverse transcription, were used as normal and internal controls, respectively. The pCMV-16K rPRL plasmid was used as a positive control. The PCR product (487 bp) was obtained as expected (B), and 16K rPRL expression was confirmed by Southern blot (C). D. 16K rPRL protein detection. Proteins from control muscle and serum were used as negative control. One microliter of serum was obtained, electrophoresed, and subjected to western blot as described in Materials and Methods. The western blot shown is representative of results obtained from four independent experiments. → ←: primer site for PCR, RT: reverse transcription, pCMV-16K rPRL: pCMV-16K rPRL-injected mice. pCMV-16K rPRL-His: pCMV-16K rPRL-His-injected mice.

**Figure 2.**
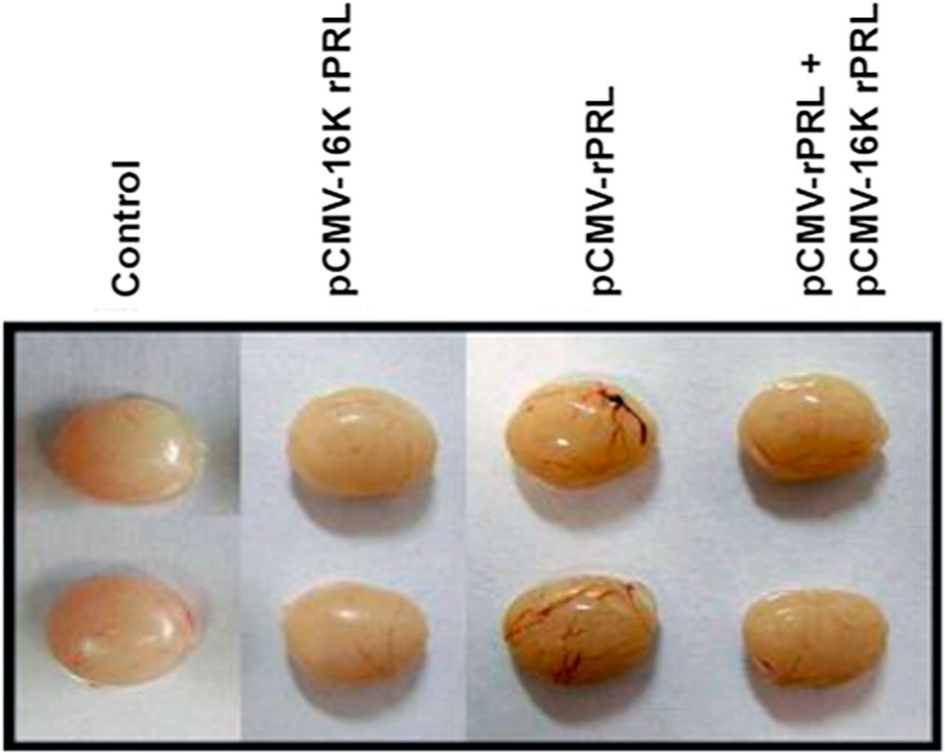
Anti-angiogenic activity of 16K rPRL. Gross morphology of the testes at 8 weeks after injection is shown. Angiogenesis induced by rPRL was inhibited by 16K rPRL.

**Figure 3.**
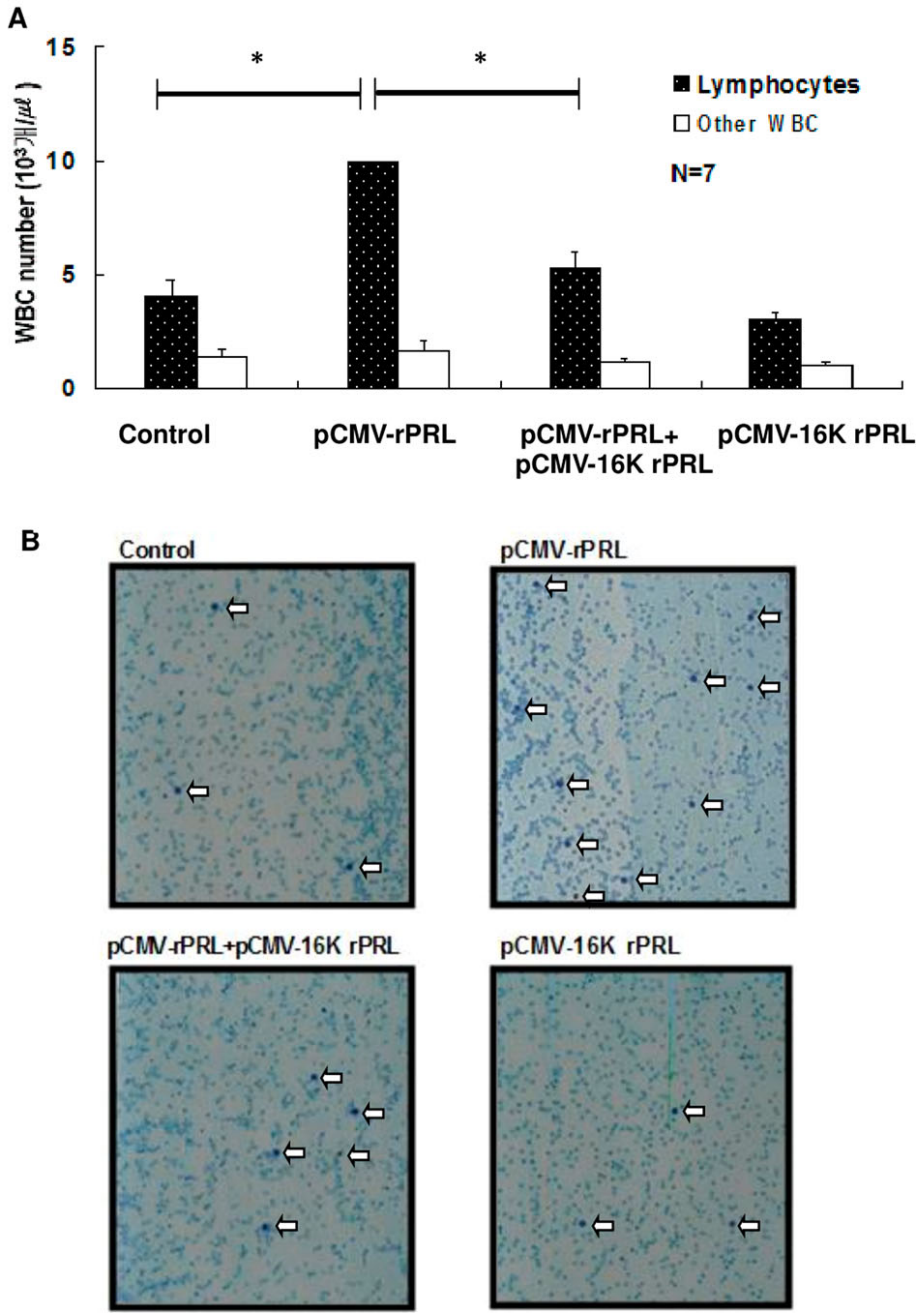
16K rPRL antagonizes the WBC proliferation induced by rPRL. A. Fresh blood samples from the tail were collected 10 days after second injection in the model shown in Figure 3. Then, lymphocyte and neutrophil cells were counted. Asterisks denote values that are significantly different from mean control values (ANOVA, * *p* < .01 compared to control).

**Figure 4.**
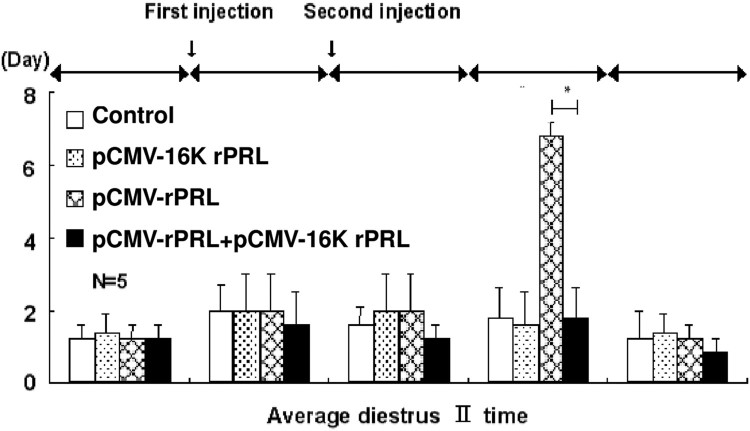
Change in the estrous cycle in 16K rPRL-expressing mice. Each stage of the estrous cycle was identified by daily examination of vaginal cytology at 9:30 AM at 100× magnification (*n* = 5). The first injection was carried out after confirming two normal estrous cycles, and the second injection followed 4 days later. Note that diestrus was extended after the second injection. Asterisks denote values that are significantly different from the mean control value (Student's *t*-test, *P* < .01). Values shown are means ± standard deviations.

**Figure 5.**
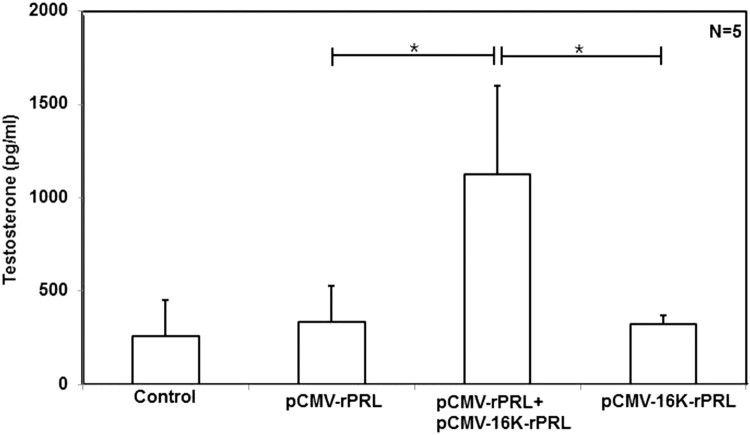
Testosterone level. A. Fresh blood samples from the tail were collected 10 days after the second injection in the model shown in⁠ Figure 3. Then, testosterone levels were measured as described in Materials and Methods. Asterisks denote values that are significantly different from mean control values (ANOVA, *p < .01 compared to control).

ICR mice at 2 months of age were purchased from the Daehan Animal Center and maintained with 14 h light, 10 h dark illumination at 23°C, and food and water *ad libitum*. Plasmid pCMV-16k rPRL for injection was purified using a slightly modified alkaline lysis method (Ko et al. 2003; Lee et al. 2006). To measure PRL mRNA (Figure 1), muscle tissue and blood were harvested 4 days after a single injection of 200 μg pCMV-16k rPRL in 50 μl of 10% sucrose in saline. For the angiogenesis study, two injections of pCMV-rPRL or pCMV-16K rPRL or pCMV-rPRL plus pCMV-16K rPRL were made 7 days apart into the quadriceps of male mice. Testes were harvested at 8 weeks after injection (Figure 2, Table 1). For the WBC proliferation study (Figure 3), blood was harvested at 10 days after the second injection in the model shown in Figure 2. For the reproductive studies (Figure 4), the first injection of pCMV-16k rPRL (or pCMV-rPRL) was performed in 2-month-old female mice at 10:00 AM on diestrus II, and the second 7 days later. For the hormonal study, blood was harvested at 10 days after injection in the model shown in Figure 3 (Figure 5). Control mice were injected with pcDNA3 vector or vehicle. All experiments were performed at least four times if not otherwise noted, and representative results are shown.

**Table 1.** Rate of new blood vessel formation.

	Increased testes/total testes	Increased testes/total testes
Control	0/9	0.0
pCMV-16K rPRL	0/9	0.0
pCMV-rPRL	7/9	77.8
pCMV-rPRL + pCMV-16K rPRL	3/9	33.3

pCMV-16K rPRL: pCMV-16K rPRL-injected mice, pCMV-rPRL: pCMV-rPRL-injected mice. pCMV-16K rPRL + pCMV-rPRL: pCMV-16K rPRL + pCMV-rPRL-injected mice

### Construction of the gene expression vector

To generate pCMV-16K rPRL (6009 bp), a rat PRL cDNA (a kind gift from Dr. D. H. Linzer, Northwestern University) was digested with Hind III and Bgl II and cloned into the Hind III and Bam HI site of vector pcDNA3 (Invitrogen, USA), which has a CMV early promoter and a bovine growth hormone polyadenylation site (Figure 1(A)). For clear detection of 16K rPRL protein using the His tag, the pCMV-16K rPRL-His plasmid was constructed. The above rat PRL cDNA was digested with Hind III and Bgl II and cloned into the Hind III and Bam HI site of vector pcDNA4myc-His (Invitrogen, USA), which shares the common gene expression system of pCDNA3 with the exception of the additional His tag.

### Reverse transcription-polymerase chain reaction (RT–PCR) and Southern blot hybridization

RNA was purified as previously described (Ko et al. 2003; Lee et al. 2006). Briefly, muscles were homogenized with denaturing solution [4 M guanidinium thiocyanate, 25 mM sodium citrate (pH 7), 0.5% N-laurylsarcosine, 0.1 M 2-mercaptoethanol]. The homogenate was phenol/chloroform-extracted, and RNA precipitated. RNA was quantified using a UV spectrophotometer (U.V.2000, Pharmacia). The absorption ratio (A260/A280) ranged from 1.8 to 2.0. Ten micrograms of total RNA were used after quantification in duplicate. RT–PCR and Southern blot hybridization were performed as described (Cho et al. 2001). RNA was then treated with DNase I (5 U, Promega, USA) at 37°C for 10 min in order to remove genomic and transfected plasmid DNA, and reverse transcribed at 42°C using random hexamer primers and AMV reverse transcriptase (Promega, USA) in a 20 μl reaction. A mixture of oligonucleotide primers (500 ng each), dNTP, and Taq DNA polymerase (2.5 U) was added to each reaction, the total volume was brought to 100 μl with 1 × PCR buffer [10 mM Tris (pH 8.3), 50 mM KCl, 1.5 mM MgCl_2_ and 0.01% gelatin] and the sample was overlaid with light mineral oil. Amplification was performed for 30 cycles using an annealing temperature of 65°C on an Omn-E thermal cycler (Hybaid Limited, UK). For the 16 K rPRL gene, the primers were designed to generate a 487 bp PCR product. The 5′ primer sequence was 5′-GGA AAG CAG GGA CAC TCC TCC –3′ and the 3′ primer sequence was 5′-CCT TTG GCT TCA GGA TAG GCC-3′. After amplification, samples were chloroform extracted, dried, resuspended in 10 μl TE buffer (10 mM Tris, pH 8.0, 1 mM EDTA), and electrophoresed on a 1.2% agarose gel. The gel was photographed after ethidium bromide staining. The PCR products were then denatured with sodium hydroxide and transferred to Nytran filters (0.45 μm, Schleicher & Schuell, Germany) under vacuum. They were hybridized with dixogenein-labeled rPRL cDNA, blotted with anti-dixogenein-AP (1:1000) (Roche, Germany), washed, and exposed to X-ray film after blotting with CSPD substrate (Roche, Germany).

### Protein blot analysis

Tissues were removed, homogenized in 400 μl protein extraction buffer [0.1 M NaCl, 0.01 M Tris-Cl (pH 7.6), 1 mM EDTA (pH 8.0), 0.1% TritonX-100, 1 μg/ml aprotinin, 100 ng/ml phenylmethylsulfonyl fluoride], and centrifuged four times. The homogenates were then mixed with an equal volume of 2× SDS-loading buffer [100 mM Tris-Cl (pH 6.8), 200 mM DTT, 4% SDS, 0.2% BPB, 20% glycerol], placed in boiling water for 10 min, and centrifuged. The supernatants were transferred to fresh tubes. Samples of each extract containing 10 μg protein (10 μg) were heated at 70°C for 10 min, electrophoresed on a 12% acrylamide gel and transferred onto Nytran filters in 1× transfer buffer (39 mM glycine, 48 mM Tris base, 0.037% SDS, 20% methanol). The blots were incubated overnight in blocking solution (5% nonfat dried milk, 0.02% sodium azide, 0.02% Tween) with shaking at 4°C, followed by exposure to primary His antibody (1:3000) (Amersham Pharmacia Biotech, USA) overnight. They were washed in milk-TBS-Tween for 30 min and incubated with secondary anti-rabbit Ig (1:7500) (Sigma Aldrich, USA) in azide-free blocking solution [5% nonfat dried milk, 150 mM NaCl, 50 mM Tris-Cl (pH 7.5)] for 2 h. The secondary antibody was detected with an ECL kit (Amersham Pharmacia Biotech, USA).

### Blood cell counts and hormone measurement

For blood cells counts, fresh tail blood containing anticoagulant was collected; 0.5 μl was smeared onto a slide, stained with Wright's staining solution, and then counted under a microscope (Olympus IX-70, Japan). WBCs were recognized by their nuclear morphology and size. Average values for WBC numbers were obtained from at least five mice in each group, each of which was counted in quadruplicate. For the angiogenesis studies, excised testes from injected and control mice were examined for gross appearance. They were immediately imaged using a digital camera (DSC-F717, Sony, Japan), classified, and the number of new blood vessels was counted. For the hormone measurement, T level was determined by RIA. The sensitivity of Tassay was about 3.9 pg/tube. The intra- and inter-assay coefficients of variation were approximately 5.1% and 7.8%, respectively.

## Results

### Intramuscular expression of the 16K rPRL gene

In initial studies we asked whether intramuscular injection was an effective means of expressing 16K rPRL in mice. Using RT–PCR and Southern blot analysis, 16K rPRL mRNA was detected in mouse muscle after injection of the pCMV-16K rPRL construct, and a PCR product of 487 bp was obtained (Figure 1(B)). Subsequent hybridization with labeled rPRL cDNA confirmed that the PCR product was derived from 16K rPRL mRNA (Figure 1(C)). The 16K rPRL was detected successfully by western blot using a His antibody in sera from mice that had been injected with the pCMV-16K rPRL plasmid containg the additional His tag (Figure 1(D)). By this approach, 16K rPRL mRNA and protein were successfully expressed in muscle and secreted into the serum.

### Anti-angiogenic activity of 16K rPRL in the testis

Although there is controversy surrounding the action of PRL in angiogenesis in the region of the hypothalamus-pituitary, our previous study using rat and mouse PRL revealed new blood vessels containing abundant red blood cells on the testes (Ko et al. 2003; Lee et al. 2006). In this study, we observed anti-angiogenesis activity in the testis when pCMV-16K rPRL combined with pCMV-rPRL was injected, whereas rPRL alone induced angiogenesis (Figure 2). The rate of blood vessel formation in the testis was 33.3% in the pCMV-rPRL plus pCMV-16K rPRL group, compared with 77.8% in the pCMV-rPRL group (Table 1).

### Blockade of WBC proliferation by 16K rPRL

Since PRL is known as a co-mitogen for T and B cells and increased WBC proliferation was observed in rat and mouse PRL-overexpressing mice (Ko et al. 2003; Lee et al. 2006), 16K rPRL activity with regard to WBC proliferation was investigated. Proliferation of WBCs was reconfirmed after 10 days of second injection in the pCMV-23K rPRL. However, 16K rPRL combined with rPRL blocked WBC proliferation, which was induced by rPRL alone (Figure 3(A)). On histological examination no conspicuous differences were observed between the experimental groups other than the number of WBCs (Figure. 3(B)).

### Effect of 16K rPRL on reproductive function

In females, the role of PRL in ovarian function is contested⁠. In our previous studies, we observed that ectopically expressed PRL extended the estrous cycle, especially the diestrus stage (Ko et al. 2003; Lee et al. 2006). Initially we reconfirmed this extended diestrus in mice injected with pCMV-rPRL. Vaginal cytology was examined daily at 9:30 A.M. after plasmid injection in female mice. Importantly, 16K rPRL combined with rPRL blocked the extension of the diestrus stage that was observed in the group expressioning rPRL alone (Figure 4). In males, the serum T level was increased in the pCMV-rPRL plus pCMV-16K rPRL group, whereas the T level was unchanged in the pCMV-rPRL or in the pCMV-16K rPRL groups compared to control (Figure 5).

## Discussion

We have shown that direct injection of pCMV-16K rPRL plasmid into mouse muscle leads to the appearance of 16K rPRL mRNA and protein, and has a variety of consequences, including changes in testicular blood vessel, WBC number, estrous cycle, and testosterone production. Until now, the role of 16K PRL has been poorly understood *in vivo* whereas PRL has been demonstrated to be required during lactation and reproduction. Our findings indicate that 16K PRL isoform has integral functions in angiogenesis of the testis, WBC proliferation, and reproduction, in addition to its already known function in angiogenesis and endothelial cell proliferation (Bernard et al. 2015).

Because the anti-angiogenic activity of 16K PRL is already known (Clapp et al. 1993), we reinvestigated that of 16K rPRL using our research protocol. The angiogenic activity of PRL was demonstrated using the same research protocol as previously used (Ko et al. 2003; Lee et al. 2006). PRL induced angiogenesis in the testis 5 weeks after plasmid injection with branching on the surface of the testis (Ko et al. 2003; Lee et al. 2006), although it has been reported that intact PRL did not play a stimulatory role in angiogenesis (Ferara et al. 1991). Compared to the angiogenic role of PRL, 16K rPRL reduced angiogenesis in the testis when pCMV-16K rPRL combined with pCMV-rPRL was injected into mice (Figure 2 and Table 1). Histological examination of cross-sections of the testes revealed the same pattern. The size and morphology of the seminiferous tubules were no different from those of control mice (data not shown). Angiogenesis is an important aspect of many physiological processes (Hanahan and Folkman 1996) as well as of pathological conditions such as tumor growth and metastasis (Folkman 1995; Bernard et al. 2015). Recently it was reported that enhanced 16K PRL is associated with postpartum cardiomyopathy (Hilfiker-Kleiner et al. 2007). Decreased serum levels of 16K PRL in patients with diabetes mellitus could contribute to the development and progression of diabetic retinopathy (Triebel et al. 2009). The observed anti-angiogenic activity of 16K rPRL in the testis suggests that 16K rPRL has an integral role in male reproductive physiology or pathophysiology. It is important to note that it is not easy to evaluate these effects in a 16K PRL or PRL *in vivo* model because transgenic animals overexpressing 16K PRL alone have not yet been generated and transgenic animals overexpressing PRL did not reveal any induced angiogenesis (Wennbo et al. 1997). In fact, most angiogenesis studies have been accomplished using semi-*in vivo* models such as Matrigel plug assay, corneal angiogenesis assay, chicken chorioallantoic membrane (CAM) assay, and hindlimb ischemia assay (Tahergorabi and Khazaei, 2012). Therefore our animal model will prove useful for the study of the anti-angiogenesis or angiogenesis efects of 16K PRL and PRL. Furthermore, it will be a useful model for the study of *in vivo* angiogenesis or anti-angiogenesis effects of secreted protein hormones if the target organ or tissue is known.

As to the role of 16K PRL in WBC proliferation, nothing has been known. In contrast, the *in vitro* mitogenic effects of PRL on T and B cells of human or murine origin (Russell et al. 1984; Bernton et al. 1988; Clevenger et al. 1990), as well as on NK (natural killer) cells and macrophages (Bernton et al. 1988) are well known. Our previous study revealed that induced PRL stimulates WBC proliferation (Ko et al. 2003; Lee et al. 2006). In this study, we revealed that induced 16K PRL blocked the stimulated WBC proliferation, revealing an antagonist activity (Figure 3). With regard to cell proliferation, it is known that 16K PRL inhibits activation of the MAPK pathway (D’Angelo et al. 1999), induces cell cycle arrest of endothelial cells (Tabruyn et al. 2005), induces programmed cell death in endothelial cells (Martini et al. 2000) through nuclear factor-kB activation (Tabruyn et al. 2003), and inhibits tumor growth in mice (Bentzien et al. 2001). In addition, it is known that PRL is required for mitogenesis during interleukin 2-driven T lymphocyte proliferation (Clevenger et al. 1990, 1991). PRL also acts as a survival factor during periods of stress (Kant et al. 1992) and inhibits glucocorticoid-induced apoptosis (Fletcher-Chiappini et al. 1993; Witorsch et al. 1993). PRL overexpression studies⁠ revealed that PRL induced cell proliferation in prostate hyperplasia (Kindblom et al. 2003). However, controversial reports have also been published. PRL-deficient mice revealed that PRL does not play an indispensable role in primary lymphocyte development and homeostasis (Horseman et al. 1997) and PRL receptor-deficient mice showed no alteration in their content of thymic or splenic cells (Bouchard et al. 1999). Collectively, the precise role and mechanism of 16K PRL or PRL must be investigated further. Our results only suggest that 16K rPRL plays an integral role in WBC proliferation. One important fact is that the action of 16K PRL in angiogenesis, tumorigenesis, and disease is not effected through PRL-R signaling. The binding partner of 16K PRL is plasminogen activator inhibitor-1 (PAI-1). PAI-1 inhibits tissue-type plasminogen activator (tPA) and urokinase-type plasminogen activator (uPA). 16K PRL impairs tumor vascularization through the PAI-1 complex (Bajou et al. 2014). Thus more studies are needed to ascertain whether the PAI-1 complex is needed for the function of 16K PRL in WBC proliferation.

Although PRL is known well to participate in regulation of reproduction (Leong et al. 1983), osmoregulation (Neill 1988), and immununomodulation (Bole-Feysot et al. 1998), relatively little is known at the molecular level, because PRL-transgenic (Wennbo et al. 1997; Kindblom et al. 2003), PRL-deficient (Horseman et al. 1997), or PRL-R-deficient (Bouchard et al. 1999) studies have revealed no conspicuous results. In *in vivo* animal studies in females, it was reported that PRL-deficient mice had irregular estrous cycles and did not become pregnant when mated to stud males (Horseman et al. 1997). Our previous study using a transient gain-of-function animal model revealed that induced PRL extended the estrous cycle, especially in the diestrus stage (Ko et al. 2003; Lee et al. 2006). In females, the function of 16K PRL has recently been understood. The role of 16K PRL in postpartum cardiomyopathy (Hilfiker-Kleiner et al. 2007), and the onset of preeclampsia (Gonzalez et al. 2007) is understood to a limited extent. In our study, data revealed that 16K PRL blocked the extended diestrus stage of the estrous cycle (Figure 4). Thus, our study suggests that 16K PRL or PRL has an integral function in the estrous cycle, an essential part of reproduction. In males, PRL receptor mRNA has been observed in the testis as well as the liver, and prostate gland (Boutin et al. 1988). With regard to PRL binding, we obtained evidence for binding to Leydig cells within the testes using PRL-EGFP (Ko et al. 2003), and found that PRL induces new blood vessel formation (Ko et al. 2003; Lee et al. 2006). In our study, 16K PRL blocked the new blood vessel formation induced by PRL (Figure 2). New blood vessel formation could potentially be associated with the two main functions of the testis, steroidogenesis and spermatogenesis. We found, unexpectedly, that 16K PRL combined with PRL induced testosterone level (Figure 5), suggesting that steroidogenesis is influenced by 16K PRL and PRL.
